# Pretreatment Contrast-Enhanced Computed Tomography Radiomics for Prediction of Pathological Regression Following Neoadjuvant Chemotherapy in Locally Advanced Gastric Cancer: A Preliminary Multicenter Study

**DOI:** 10.3389/fonc.2021.770758

**Published:** 2022-01-07

**Authors:** Kun Xie, Yanfen Cui, Dafu Zhang, Weiyang He, Yinfu He, Depei Gao, Zhiping Zhang, Xingxiang Dong, Guangjun Yang, Youguo Dai, Zhenhui Li

**Affiliations:** ^1^ Department of Radiology, Yunnan Cancer Hospital, Yunnan Cancer Center, the Third Affiliated Hospital of Kunming Medical University, Kunming, China; ^2^ Department of Radiology, Shanxi Province Cancer Hospital, Shanxi Medical University, Taiyuan, China; ^3^ Department of Gastrointestinal Surgery, Sichuan Province Cancer Hospital, University of Electronic Science and Technology of China, Chengdu, China; ^4^ Department of Gastric and Surgery, Yunnan Cancer Hospital, Yunnan Cancer Center, the Third Affiliated Hospital of Kunming Medical University, Kunming, China

**Keywords:** locally advanced gastric cancer, neoadjuvant chemotherapy, pathological response, CECT, radiomics

## Abstract

**Background:**

Sensitivity to neoadjuvant chemotherapy in locally advanced gastric cancer patients varies; however, an effective predictive marker is currently lacking. We aimed to propose and validate a practical treatment efficacy prediction method based on contrast-enhanced computed tomography (CECT) radiomics.

**Method:**

Data of l24 locally advanced gastric carcinoma patients who underwent neoadjuvant chemotherapy were acquired retrospectively between December 2012 and August 2020 from three different cancer centers. In total, 1216 radiomics features were initially extracted from each lesion’s pretreatment portal venous phase computed tomography image. Subsequently, a radiomics predictive model was constructed using machine learning software. Clinicopathological data and radiological parameters of the enrolled patients were collected and analyzed retrospectively. Univariate and multivariate logistic regression analyses were performed to screen for independent predictive indices. Finally, we developed an integrated model combining clinicopathological predictive parameters and radiomics features.

**Result:**

In the training set, 10 (14.9%) patients achieved a good response (GR) after preoperative neoadjuvant chemotherapy (n = 77), whereas in the testing set, seven (17.5%) patients achieved a GR (n = 47). The radiomics predictive model showed competitive prediction efficacy in both the training and independent external validation sets. The areas under the curve (AUC) values were 0.827 (95% confidence interval [CI]: 0.609–1.000) and 0.854 (95% CI: 0.610–1.000), respectively. Similarly, when only the single hospital data were included as an independent external validation set (testing set 2), AUC values of the models were 0.827 (95% CI: 0.650–0.952) and 0.889 (95% CI: 0.663–1.000) in the training set and testing set 2, respectively.

**Conclusion:**

Our study is the first to discover that CECT radiomics could provide powerful and consistent predictions of therapeutic sensitivity to neoadjuvant chemotherapy among gastric cancer patients across different hospitals.

## Introduction

Gastric cancer (GC) is a global malignancy with high rates of incidence and mortality (1). In recent years, the incidence of GC has increased in China (2). Approximately 80% of GC patients have progressed to the locally advanced stage by the time of initial diagnosis (3). Neoadjuvant chemotherapy is one of the recommended treatment options for locally advanced GC (LAGC), according to the current China Society and Clinical Oncology and National Comprehensive Cancer Network (NCCN) guidelines (3, 4). Neoadjuvant chemotherapy can improve radical resection rates (5) and reduce the risk of postoperative recurrence and metastasis.

A previous study demonstrated that pathological complete response (pCR) after preoperative chemotherapy is correlated with better prognosis (6). However, even with the most effective neoadjuvant chemotherapy, a good response (GR) (TRG0/1) is achieved in only approximately 20%–40% of LAGC patients, according to previous large multicenter, randomized controlled studies (7, 8). This suggests that at least 60% of LAGC patients are non-responders, who may be experiencing unnecessary toxicity and side effects of chemotherapeutic agents, suboptimal surgical timings, and disease progression. Currently, the lack of an effective clinical predictive method is resulting in low response rates to chemotherapy during the preoperative phase in LAGC patients (9).

Radiomics is an emerging technology that involves the extraction of quantitative features from medical images, such as computed tomography (CT) and magnetic resonance imaging (MRI). Radiomics features contain extensive numerically invisible image messages that are closely associated with the biobehavioral, microenvironmental, and genetic expressions of primary tumors (10, 11). Radiomics may serve as a non-invasive indicator for the outcome of therapeutic sensitivity and prognosis, which may facilitate the development of an individualized treatment plan, such as upfront radical surgery, radiotherapy, and palliative care. Previous studies have revealed that radiomics have predictive value for outcomes of patients with LAGC who are undergoing neoadjuvant chemotherapy (12–14). However, these studies only investigated small samples, were single-centered, and lacked independent external validation, which is insufficient for clinical decision-making and transformation. Moreover, the accuracy of the prediction models for LAGC patients required further improvement. Therefore, our study aimed to validate the generalizability, validity, and applicability of contrast-enhanced CT (CECT)-based radiomics in predicting pathological tumor regression following neoadjuvant chemotherapy in patients with locally advanced gastric cancer.

## Materials and Methods

This was a multi-centered retrospective study using data from databases of three provincial cancer hospitals in China. Local ethics committees approved the multicenter retrospective study. We adhered to the guidelines of the Helsinki Declaration. Informed consent was waived because we could not contact patients who had been discharged from the hospital.

### Patients

We collected data of 124 patients with clinical stage II/III LAGC who received neoadjuvant chemotherapy between December 2012 and August 2020. All subjects were selected according to the following inclusion and exclusion criteria.

Inclusion criteria: (a) histologically proven gastric adenocarcinoma by endoscopic biopsy histopathology; (b) clinical stage T3−T4 and N+ or N− without evidence of distant metastasis, according to the Eighth Edition of the American Joint Committee on Cancer guidelines (15); (c) neoadjuvant chemotherapy according to the SOX regimen (S‐1 + oxaliplatin) was performed for two to six 3-week cycles; (d) underwent radical gastrectomy and histological grading data were reviewed by a pathologist; (e) received CECT examination within one week before neoadjuvant chemotherapy.

Exclusion criteria were: (a) thickness of the lesion was < 0.5 cm (because the regions of interest (ROI) were difficult to outline precisely); (b) the degree of gastric filling was insufficient, which resulted in unclear locations and boundaries of primary lesions; (c) patients who received both neoadjuvant chemotherapy and radiotherapy; and (d) extreme respiratory motion artifacts.

All clinical and histopathological data of the enrolled LAGC patients were collected retrospectively, including age, sex, body mass index (BMI), primary tumor site (funds of stomach, body of stomach, or autrum of stomach), tumor differentiations, pretreatment clinical stage, serum CA19-9, serum CEA, and preoperation clinical stage.

### Neoadjuvant Chemotherapy

All enrolled patients were treated with a SOX-based chemotherapy regimen in three different hospitals before surgery. Details of the neoadjuvant chemotherapy regimen were as follows. Oxaliplatin was injected through an intravenous access at 130 mg/m^2^ over 2 hours on day 1 for LAGC patients. Subsequently, patients were administered one dose of 80 mg S-1 once a day orally, for 14 days per cycle. All patients received 2–6 cycles of neoadjuvant chemotherapy before surgery until disease progression or intolerable toxicity.

### CT Scan Protocol

CECT images of all patients were obtained within 7 days of commencement of any anti-cancer treatment. The CT scanning protocol was as follows. Patients fasted for 8–12 hours and drank 800–1200 mL of water 15 minutes before the CT examination to distend their stomach. Before enhancement, a plain scan was performed to locate the primary lesion and determine the scanning range. The iodine contrast material was injected into the arm vein at an injection rate of 3.0–3.5 mL/s using a high-pressure injector. The total injection volume of contrast media was 80−100 mL, based on 1–1.5 mL/kg of body weight. Subsequently, 15–20 mL of saline flush were injected at the same rate. Portal venous images were acquired 55–65 sec after injection of the contrast agent. Detailed CT scanning parameters are provided in **Supplementary Table 1**.

### Response Evaluation Criteria

Response to neoadjuvant chemotherapy was based on postoperative pathology results. All resection specimens were independently assessed by a pathologist, who had > 20 years of experience and was blinded to other clinical data. Tumor regression grading criteria were selected according to the latest NCCN guideline (version 4, 2021) (4). Tumor regression grade (TRG) 0 was defined as no viable tumor cell in the primary lesion or lymph nodes, TRG 1 was defined as a single cell or rare small single group cells, TRG 2 was defined as obvious tumor cell disappearance but more than a single cell or few residual gastric adenocarcinoma cells, and TRG 3 was defined as no evident tumor regression. TRGs 0–1 were then defined as a good response (GR), and TRGs 2–3 were defined as a non-good response (non-GR).

### CT Value Measurement of LAGC

Two readers independently reviewed all the slices of portal venous images of each patient. Both were blind to the clinicopathological data of patients. They then independently manually delineated a circular ROI on the selected maximum layer of LAGC on the portal-venous images, which was necessary to avoid areas containing necrosis or air. Mean CT values of the primary lesion (CT1) were obtained. To eliminate individual differences, we measured the mean CT values of abdominal aortic calcium (CT2) on the same slice using the same measurement method. Normalized CT values were then calculated as CT1/CT2. The intraclass correlation coefficient (ICC) test was performed to assess the agreement of CT value measurements between the two reviewers. An ICC of > 0.75 was considered adequate consistency. The means of both measurements were used in the final analyses.

### Tumor Segmentation and Image Feature Extraction

Portal phase images of all LAGC patients were viewed using the ITK-SNAP software (version 3.8.0, www.itksnap.org) (16) for image analysis, which was carried out by a single radiologist with 15 years of experience in abdominal tumor imaging diagnosis. A two-dimensional ROI was selected according to relevant literature (17). The ROI was manually outlined along the margin of the LAGC lesion on the maximum layer, which was necessary to avoid areas containing necrosis or air. We selected the top 30 patients ranked by the baseline CT examination date in the test dataset again, and their ROIs were manually outlined by another radiologist separately. Before feature extraction, all images and corresponding ROIs were resampled to a voxel size of 1 × 1 × 1 mm. Then, 1216 features were acquired using the Analysis-kit software (AK, version 3.2.0, GE Healthcare), which comprised 252 first-order parameters, 14 shape parameters, 336 gray-level co-occurrence matrices, 224 gray-level run-length matrices, 224 gray-level size-zone matrices, 70 neighborhood gray-tone difference matrices, and 96 gray-level dependence matrices.

### Radiomics Feature Selection and Model Building

To remove the imbalance of the training data set, we up-sampled by repeating random cases to achieve a positive/negative sample balance. In addition, radiomics feature data were processed using the max-min normalization method. Dimensionality reduction procedures of the extracted radiomics features were performed using Pearson correlation coefficients (PCC). If the PCC of two features was higher than 0.99, one of the features was removed. Before building the model, the relief selector was used to identify relevant features. Subsequently, the support vector machine (SVM) was selected as the classifier to construct the model. We adopted 10-fold cross-validation to test the model’s predictive ability. The above radiomics feature selection and model building processes were conducted using an open-source software called FAE (FeAture explorer, version 0.3.7; https://github.com/salan668/ FAE) (18). The calibration between the model and the independent external validation dataset was assessed using a calibration curve. Subsequently, subgroup analyses were performed for sex and location of the LAGC.

### Statistical Analysis

Statistical analysis was performed using the R software (version 2.3.3, https://www.r-project.org/). For clinical-pathological parameters, quantitative variables were subjected to an independent-samples t-test. Categorical variables were analyzed using chi-square or Fisher’s exact tests. Wilcoxon rank-sum tests were performed for ranked data. Then, univariate and multivariate logistic regression analyses were carried out to identify response indicators of neoadjuvant chemotherapy in the training set. A clinical predictive model was built based on the selected clinical factors. A *p* < 0.05 was considered statistically significant. Finally, an integrated predictive model was constructed by integrating the radiomics signature with the clinical predictors, which was named the clinical-rad model.

## Results

### Basic Clinical and Histopathological Characteristics of Patients

**Figure 1:**
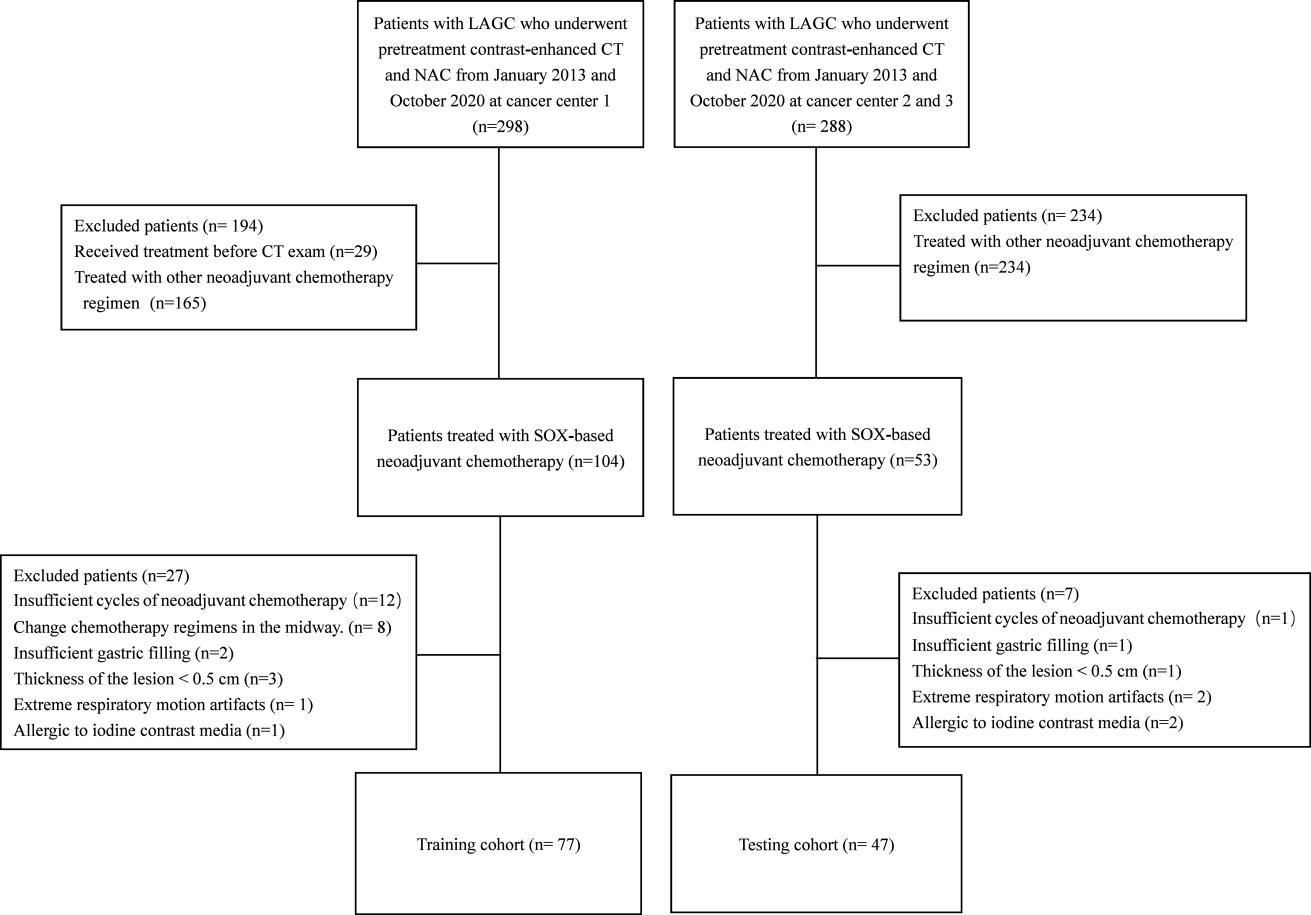
Flowchart of the inclusion and exclusion process.

We enrolled 124 locally advanced gastric carcinoma patients who underwent SOX-based neoadjuvant chemotherapy between December 2012 and August 2020. Specific inclusion and exclusion processes were shown in the flowchart in **Figure 1**. Of these, 77 patients were used to form the training set, and 47 patients were classified as the testing set. The average duration of neoadjuvant chemotherapy before surgery was approximately 2-3 cycles for both the training and testing datasets (*p* > 0.05). Ten (14.9%) patients in the training set achieved a GR after neoadjuvant chemotherapy, and seven (17.5%) patients in the testing set achieved a GR after treatment. Analyses showed that there were no significant differences in baseline clinical and histopathological characteristics between the training and testing sets, except for the sex, primary tumor site, preoperation T stage, CEA and CA19-9 levels. Furthermore, apart from preoperation T stage, preoperation M stage, and degree of differentiation of the primary lesion, no significant differences were found in clinical and histopathological data between the GR and non-GR patients in the training set. Detailed clinical and pathological information on the training and testing cohorts are shown in **Tables 1** and **2**. However, univariate logistic regression analyses showed that there were no independent predictors of response to neoadjuvant chemotherapy in the training set. Therefore, the multivariate logistic regression predictive model was not built (detailed information is shown in **Table 3**).

**Table 1:** Clinicopathological information of the 124 locally advanced gastric cancer patients.

Variable	Training cohort (n = 77)	Testing cohort (n = 47)	*P* Value
Age/years (mean ± SD)	58.36 ± 9.86	56.04 ± 10.95	0.228
Sex			0.004
Male	64 (83.1)	28 (59.6)	
Female	13 (16.9)	19 (40.4)	
BMI/kg*m^-2^ (mean ± SD)	22.85 ± 2.95	22.72 ± 3.40	0.827
Cycle of NAC	2 (2-4)	3 (2-3)	0.557
Primary tumor site			<0.001
Funds of stomach	20 (26.0)	9 (19.1)	
Body of stomach	42 (54.5)	9 (19.1)	
Autrum of stomach	15 (19.5)	29 (61.7)	
Pre-treatment T stage			0.335
T2-3	41 (53.2)	21 (44.7)	
T4	36 (46.8)	26 (55.3)	
Pre-treatment N stage			0.632
N0	13 (16.9)	5 (10.6)	
N1	15 (19.5)	13 (27.7)	
N2	24 (31.1)	15 (31.9)	
N3	25 (32.5)	14 (29.8)	
Preoperation T stage			<0.001
T0-1	10 (13.0)	8 (17.0)	
T2	4 (5.2)	10 (21.3)	
T3	57 (74.0)	10 (21.3)	
T4	6 (7.8)	19 (40.4)	
Preoperation N stage			0.979
N0	26 (33.8)	15 (31.9)	
N1	13 (16.9)	7 (14.9)	
N2	18 (23.4)	12 (25.5)	
N3	20 (25.9)	13 (27.7)	
Preoperation M stage			0.053
M0	65 (84.4)	45 (95.7)	
M1	12 (15.6)	2 (4.3)	
Differentiation			0.250
High differentiation	5 (6.5)	3 (6.4)	
Moderate differentiation	25 (32.4)	9 (19.1)	
Poor differentiation	47 (61.0)	35 (74.5)	
CEA			<0.001
Normal	50 (64.9)	13 (27.7)	
Elevated	27 (35.1)	34 (72.3)	
CA19-9			0.010
Normal	51 (66.2)	41 (87.2)	
Elevated	26 (33.8)	6 (12.8)	

BMI, body mass index; CEA, carcinoembryonic antigen; NAC, neoadjuvant chemotherapy.

**Table 2:** Comparison of the clinicopathological information of responders and non-responders in the training and two testing cohorts.

Characteristic	Training cohort			Testing cohort		
	GR (n = 10)	non-GR (n = 67)	*P* Value	GR (n = 7)	non-GR (n = 40)	*P* Value
Age/years (mean ± SD)	57 (44–57)	60 (55–65)	0.340	59 (45–65)	56 (46–66)	0.881
Sex			0.663			1.000
Male	8 (80.0)	56 (83.6)		4 (57.1)	23 (57.5)	
Female	2 (20.0)	11 (16.4)		3 (42.9)	17 (42.5)	
BMI/kg*m^-2^ (mean ± SD)	22.81 ± 2.95	23.08 ± 3.10	0.802	21.42 ± 2.13	22.95 ± 3.55	0.278
Cycles of NAC	3 (2–4)	3 (2–3.9)	0.759	2 (2–3)	2.5 (2–4)	0.092
Primary tumor site			0.082			0.775
Funds of stomach	4 (40.0)	16 (23.9)		2 (28.6)	7 (17.5)	
Body of stomach	6 (60.0)	36 (53.7)		1 (14.3);	8 (20.0)	
Autrum of stomach	0 (0.0)	15 (22.4)		4 (57.2)	25 (62.5)	
Pre-treatment T stage			0.425			0.666
T2-3	7 (70.0)	34 (50.7)		4 (57.1)	16 (40.0)	
T4	3 (30.0)	33 (49.3)		3 (42.9)	24 (60.0)	
Pre-treatment N stage			0.648			0.139
N0	1 (10.0)	12 (17.9)		1 (14.3)	4 (10.0)	
N1	3 (30.0)	12 (17.9)		3 (42.9)	10 (25.0)	
N2	2 (20.0)	22 (32.8)		3 (42.9)	12 (30.0)	
N3	4 (40.0)	21 (31.3)		0 (0.0)	14 (35.0)	
Preoperation T stage			<0.001			<0.001
T0-1	4 (40.0)	2 (3.0)		7 (100.0)	1 (2.5)	
T2	2 (20.0)	4 (6.0)		0 (0.0)	10 (25.0)	
T3	0 (0.0)	55 (82.1)		0 (0.0)	10 (25.0)	
T4	3 (30.0)	6 (8.9)		0 (0.0)	19 (47.5)	
Preoperation N stage			0.567			0.302
N0	5 (50.0)	21 (31.3)		4 (57.2)	11 (27.5)	
N1	2 (20.0)	11 (16.4)		1 (14.3)	7 (17.5)	
N2	2 (20.0)	16 (23.9)		1 (14.3)	10 (25.0)	
N3	1 (10.0)	19 (28.4)		1 (14.3)	12 (30.0)	
Preoperation M stage			0.000			1.000
M0	2 (20.0)	57 (85.1)		7 (100.0)	37 (92.5)	
M1	8 (80.0)	10 (14.9)		0 (0.0)	3 (72.5)	
Differentiation			0.027			0.336
High differentiation	0 (0.0)	5 (7.5)		1 (14.3)	2 (5.0)	
Moderate differentiation	0 (0.0)	25 (37.3)		2 (28.6)	7 (17.5)	
Poor differentiation	10 (100.0)	37 (55.2)		4 (57.1)	31 (77.5)	
CEA			0.475			0.125
Normal	8 (80.0)	42 (62.7)		5 (71.4)	13 (32.5)	
Elevated	2 (20.0)	25 (37.3)		2 (28.6)	27 (67.5)	
CA19-9			1.000			0.049
Normal	7 (70.0)	44 (65.7)		4 (57.1)	37 (92.5)	
Elevated	3 (30.0)	23 (34.3)		3 (42.9)	3 (7.5)	
CEA combined CA19-9			0.956			0.929
Normal	9 (90.0)	56 (83.6)		6 (85.7)	38 (95.0)	
Elevated	1 (10.0)	11 (16.4)		1 (14.3)	2 (5.0)	
CT values (Hu)	92.66 ± 4.48	98.05 ± 19.44	0.432	83.59	72.36 ± 17.11	0.138
Standardized CT value	0.57 (0.49-0.62)	0.58 (0.53–0.66)	0.544	0.49	0.48 ± 0.15	0.901

BMI, body mass index; CEA, carcinoembryonic antigen; CT, computed tomography; GR, good response; NAC, neoadjuvant chemotherapy; non-GR, non-good response.

**Table 3:** Univariate and multivariate logistic regression analyses of the independent predictors of neoadjuvant chemotherapy in the training set.

Characteristic	Univariate logistic regression analyses		Multivariate logistic regression analyses	
	HR	*P* Value	HR	*P* Value
Age/years	0.95 (0.89, 1.02)	0.15	0.96 (0.85, 1.07)	0.433
Sex				
Male	reference			
Female	1.27 (0.24, 6.82)	0.778	0.45 (0.03, 7.69)	0.577
BMI/kg*m-2	1.03 (0.82, 1.29)	0.787	0.99 (0.70, 1.36)	0.892
Cycles of NAC	1.04 (0.43, 2.51)	0.925	0.82 (0.23, 2.98)	0.763
Primary tumor site				
Funds of stomach	reference			
Body of stomach	0.67 (0.17, 2.69)	0.569		0.998
Autrum of stomach	1.50 (0.37, 6.06)	0.569		0.998
Pre-treatment T stage				
T2-3	reference			
T4	2.27 (0.54, 9.51)	0.264	3.73 (0.54, 21.55)	0.181
Pre-treatment N stage				
N0	reference			
N1	0.44 (0.44, 4.38)	0.482	0.25 (0.01, 5.51)	0.379
N2	1.312 (0.25, 6.88)	0.946	1.88 (0.14, 24.55)	0.629
N3	0.477 (0.79, 2.89)	0.420	0.37 (0.04, 3.74)	0.400
Differentiation				
High differentiation	reference			
Moderate differentiation		0.999	1.000	
Poor differentiation		0.998	1.000	
CEA				
Normal	reference			
Elevated	0.30 (0.47, 12.11)	0.296	3.52 (0.10, 127.59)	0.492
CA19-9				
Normal	reference			
Elevated	1.22 (0.29, 5.17)	0.787	0.95 (0.02, 53.79)	0.980
CEA combined CA19-9				
Normal	reference			
Elevated	1.85 (0.48, 7.16)	0.373	0.55 (0.01, 46.56)	0.789
CT values (Hu)	0.99 (0.95, 1.02)	0.427	0.97 (0.91, 1.04)	0.387
Standardized CT value	0.76 (0.00, 151.24)	0.920	0.42 (0.00, 24902.12)	0.887

BMI, body mass index; CEA, carcinoembryonic antigen; CT, computed tomography; NAC, neoadjuvant chemotherapy.

### Portal Venous CT Values of LAGC

The ICC of the CT value measurements in the training set, testing set, and testing set 2 were 0.807 (95% confidence interval [CI]: 0.713–0.873), 0.784 (95% CI: 0.642–0.874), and 0.914 (95% CI: 0.803–0.964), respectively. The CT values were not significantly different between the GR and non-GR patients in the training group or the independent external testing group (*p*s = 0.432 and 0.138, respectively). However, there was a significant difference between these groups in testing set 2 (*p* = 0.043). There were no significant differences in normalized CT values between GR or non-GR patients in any of the cohorts (details are shown in **Table 2**).

### Radiomics Feature Screening and Predictive Model Building

**Figure 2:**
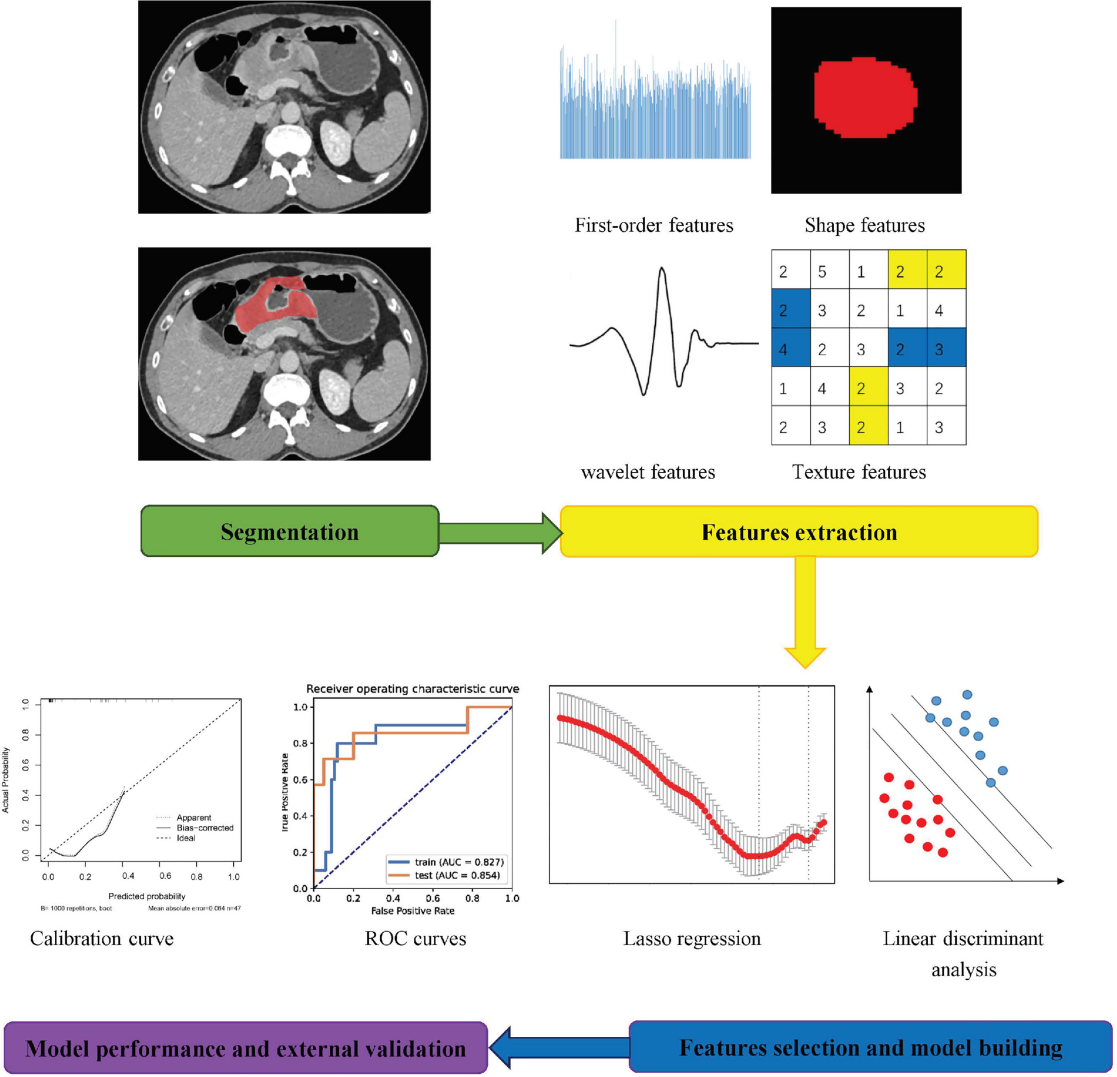
Workflow of the radiomics feature extraction and analysis process.

In total, 1216 radiomics features of each lesion were extracted from the portal venous images. ICCs were 0.58-83 for all extracted radiomics features between the two radiologists. After balancing, normalizing, and preprocessing the radiomics features, eight radiomics features were selected to establish a radiomics model to predict neoadjuvant chemotherapy efficacy using an SVM classifier. The workflow of the radiomics feature extraction and model building processes are shown in **Figure 2**.

### Performance and External Independent Validation of the Models

**Figure 3:**
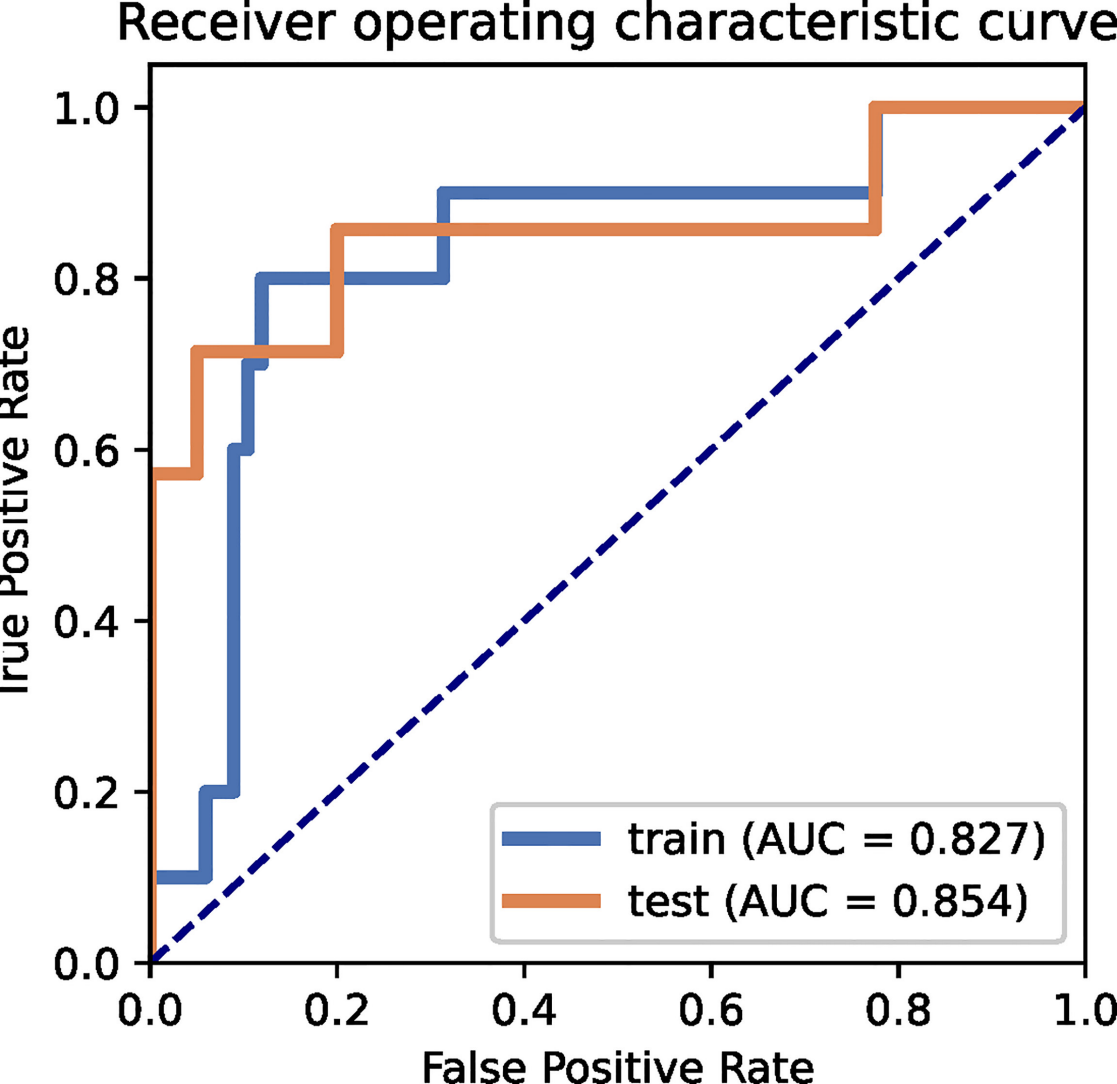
ROC curves for the radiomics predictive model of the training and external independent validation sets.

The performance of the radiomics models for predicting pathological regression following neoadjuvant chemical treatment in locally advanced gastric carcinoma is shown in **Table 4**. Receiver operating characteristic (ROC) curves of the models for the training and independent external validation cohorts are shown in **Figure 3**. In the training set, radiomics predictive efficacy achieved an area under the curve (AUC) value of 0.827 (95% CI: 0.609–1.000). A similar performance was revealed in the independent external validation cohort, with an AUC of 0.854 (95% CI: 0.610–1.000). The calibration curve result revealed a satisfactory fit between the external independent validation dataset and the radiomics predictive model (**Supplementary Figure 1**). To avoid selection bias and increase persuasive power, we selected another dataset from a single cancer center as an additional independent external validation set to test the same classifier. The AUCs of the radiomics predictive model in the training and testing cohorts were 0.827 (95% CI: 0.650–0.952) and 0.889 (95% CI: 0.663–1.000), respectively.

**Table 4:** Performance of the radiomic models in predicting the response to neoadjuvant chemotherapy of locally advanced gastric cancer in the training set and testing set.

	Training set	Testing set
AUC (95% CI)	0.827 (0.609–1.000)	0.854 (0.610–1.000)
Accuracy	0.870	0.915
sensitivity	0.800	0.714
specificity	0.881	0.950
PPV	0.500	0.714
NPV	0.967	0.950

### Subgroup Analyses of the Radiomics Predictive Model in the Training and Independent External Validation Sets

Subgroup analyses were carried out for sex and primary lesion site of the LAGCs. For the sex subgroups, AUCs of the radiomics predictive model were 0.962 (95% CI: 0.900–1.000) and 0.815 (95% CI: 0.630–0.967) for the male patients in the training and testing datasets, respectively. For the female patients, the AUCs of the radiomics predictive model achieved 0.864 (95% CI: 0.637–1.000) and 0.882 (95% CI: 0.706–0.941) for the training and testing cohorts, respectively. Subgroup analysis of the primary lesion location showed AUCs of the radiomics predictive model for the training and testing sets of 0.859 (0.438–1.000) and 0.857 (95% CI: 0.500–1.000), respectively, for stomach cancers in the upper regions (including the fundus and cardia of the stomach). Similarly, AUCs of the radiomics predictive model for the training and testing sets were 0.892 (95% CI: 0.663–1.000) and 0.812 (95% CI: 0.639–0.973), respectively, for stomach cancers in the middle and lower portion (included the corpus and antrum of the stomach). Detailed information is provided in **Supplementary Tables 2**, **3**.

## Discussion

Predicting the sensitivity of preoperative chemotherapy in patients with LAGC is essential for developing a precise and personalized therapy plan. We aimed to validate pretreatment CECT radiomics features of primary lesions to predict pathological regression after preoperative chemotherapy in patients with locally advanced gastric carcinomas. In our study, AUC values were 0.854 and 0.827 for the training and external independent testing cohorts, respectively. To further validate the model, we selected single-center data as an additional independent external validation set. The performance of the radiomics predictive models was similar, with AUC values of 0.827 in the training cohort and 0.889 in the external validation cohort, respectively.

Because of poor surgical resection rates and the high risk of disease relapse in patients with LAGC, neoadjuvant therapy was introduced as a treatment. However, there remains little consensus on the best preoperative therapy protocol for LAGC patients (19). Decisions on whether to offer neoadjuvant chemoradiotherapy, neoadjuvant chemotherapy, or direct surgical removal for LAGC patients remain unclear and controversial (20, 21). One of the main attributing factors for this is that response of LAGCs to neoadjuvant drugs varies among patients. Accurately detecting patients who are non-responsive to neoadjuvant chemotherapy and offering alternative effective treatment approaches are crucial (22). This will enable patients to avoid the side effects of chemotherapy drugs, reduce the risk of metastasis before surgery, improve prognosis, and reduce medical costs. However, there is a need for a non-invasive predictor of the neoadjuvant chemotherapeutic sensitivity for LAGC before treatment (23).

Numerous researchers have studied pretreatment CT radiomics features extensively to explore their ability to predict the efficacy of neoadjuvant chemotherapy in LAGC. However, AUCs for previous radiomics prediction models have varied from 0.70 to 0.82, depending on the treatment protocol and response evaluation criteria used (12–14, 24). Moreover, all studies lacked independent external validation, which is crucial for translating into clinical practice (25). Thus, we aimed to validate the performance of radiomics features from portal venous-phase CECT images to predict neoadjuvant chemotherapy response in LAGC patients across different hospitals. Our results showed that the predictive model demonstrated excellent agreement using CECT-based radiomics analysis across three different cancer hospitals, each achieving AUCs of > 0.80. Therefore, we concluded that CECT-based radiomics signatures may serve as a reliable and universal predictor for neoadjuvant chemotherapy response in LAGC patients.

Furthermore, although there were differences in sex and primary tumor site of LAGC patients in the training and independent external validation sets, our subgroup analyses revealed that it did not significantly affect the predictive accuracy of the radiomics model. which meant that the sex and primary tumor site of LAGC patients would not influence the performance of the pretreatment CECT-based radiomics models. The radiomics predictive model we built could be generalized to all LAGC patients.

To the best of our knowledge, our study is the first multicenter study to explore the clinical application value of pretreatment CECT radiomics in predicting the efficacy of preoperative chemotherapy in LAGC patients. The performance of our predictive model was comparable to that of Sun et al. (14) and superior to those of other previous studies. This may be because we only enrolled patients who had received SOX-based neoadjuvant chemotherapy. Recent evidence has indicated superiority or noninferiority of SOX to other neoadjuvant chemotherapy regimens and postoperative chemotherapy, based on R0 resection and disease-free survival rates (26, 27). Neoadjuvant SOX regimens may be offered as one of the most effective standardized neoadjuvant chemotherapy protocols for LAGC in the future. However, the literature suggests that adverse events and even serious adverse events can occur following the SOX regimen, which should not be ignored (26). Therefore, there is an urgent need and clinical value in accurately detecting LAGC patients who are sensitive to SOX-based neoadjuvant chemotherapy. Our findings have positive implications for individualized treatment plans. We selected postoperative pathological TRG as the termination response evaluation criteria, according to NCCN guidelines. TRG may be more accurate and suitable than other morphological response evaluation criteria because gastric filling degree can influence tumor size (28). Furthermore, a main pathological regression after neoadjuvant chemotherapy in LAGC patients indicated a good prognosis (29, 30). So we defined TRG 0–1 as GR and TRG 2–3 as non-GR. Moreover, a previous study showed that CT values of LAGC may be a non-invasive indicator of blood supply, and LAGCs with good blood supply may be more sensitive to neoadjuvant chemotherapy (31). However, we did not find differences in the CT values of primary tumors in portal venous phase images between responders and non-responders, except for in cohort 2. This may be due to the different administration routes used for neoadjuvant chemotherapy agents. Secondly, some LAGC lesions had non-uniform density in the portal venous phase CT images. Furthermore, manual measurement inevitably introduced errors. Finally, a model combining clinical and imaging data was not built because we did not identify any reliable clinical predictors of neoadjuvant chemotherapy for LAGC.

Our study also had other limitations. A major limitation is that our sample size was small, which was because a standardized neoadjuvant regimen had not yet been established, and we only selected patients who had undergone SOX-based neoadjuvant chemotherapy. In addition, there were differences in CT scanning parameters and image post-processing procedures between hospitals because the study was retrospective. Finally, our radiomics predictive model was based on single portal venous phase CECT images and thus did not include the arterial and/or delayed phase.

## Conclusion

For the first time, our findings suggest that radiomics features of CECT images may perform consistently in predicting the pathological response to neoadjuvant chemotherapy in LAGC patients from different hospitals. The model would be used as a reliable and universal tool for developing precision treatment plans for LAGC patients in the future.

## Data Availability Statement

The raw data supporting the conclusions of this article will be made available by the authors, without undue reservation.

## Ethics Statement

The studies involving human participants were reviewed and approved by the Local ethics committees of Yunnan Cancer Hospital, Sichuan Cancer Hospital and Shanxi Cancer Hospital. Written informed consent for participation was not required for this study in accordance with the national legislation and the institutional requirements. Written informed consent was not obtained from the individual(s), nor the minor(s)’ legal guardian/next of kin, for the publication of any potentially identifiable images or data included in this article.

## Author Contributions

ZL, YD, GY, and XD contributed to the study design. ZL and YD conducted the scientific literature search and collated and summarized the relevant studies. ZL, KX, YC, DZ, WH, YH, and ZZ were involved in data collection. DG, GY, XD, and KX analyzed and interpreted the data. KX and YC wrote the initial draft. The final draft of the report was approved by all authors.

## Funding

This study was funded by research grants from the National Natural Science Foundation of China [82001986, 82001789], the Applied Basic Research Projects of Yunnan Province, China, Outstanding Youth Foundation [202101AW070001], the Applied Basic Research Projects of Yunnan Province, China [2019FE001-083, 2019FE001-084, 202001AY070001-240, 202001AY070001-242], and Yunnan digitalization, Development and Application of Biotic Resource [202002AA100007].

## Conflict of Interest

The authors declare that the research was conducted in the absence of any commercial or financial relationships that could be construed as a potential conflict of interest.

## Publisher’s Note

All claims expressed in this article are solely those of the authors and do not necessarily represent those of their affiliated organizations, or those of the publisher, the editors and the reviewers. Any product that may be evaluated in this article, or claim that may be made by its manufacturer, is not guaranteed or endorsed by the publisher.

## Abbreviations

AUC, areas under the curve; CEA, carcinoembryonic antigen; CECT, contrast-enhanced computed tomography; CI: confidence interval; CT, computed tomography; GR, good response; GC, gastric cancer; LAGC, locally advanced gastric cancer; non-GR, non-good response; NCCN, National Comprehensive Cancer Network guideline; NAC, neoadjuvant chemotherapy; TRG, tumor regression grade.
