# Supplementary material for: Pretreatment Contrast-Enhanced Computed Tomography Radiomics for Prediction of Pathological Regression Following Neoadjuvant Chemotherapy in Locally Advanced Gastric Cancer: A Preliminary Multicenter Study
Source: Front Oncol. 2022 Jan 7;11:770758. doi: 10.3389/fonc.2021.770758 (PMC8777131; doi:10.3389/fonc.2021.770758)
Supplement: Supplementary file 2 [file Table_1.docx]

**Supplementary Table 1. Detailed CECT scanning protocol and the corresponding number of patients for the three different cancer hospitals.**

| **Hospital** | **Tube voltage (kVp)** | | **tube current** | **Reconstruction image thickness (mm)** | | | | | **rotation time/s** | **CT machine type** | | | |
| --- | --- | --- | --- | --- | --- | --- | --- | --- | --- | --- | --- | --- | --- |
|  | 120 | 100 |  | 0.625 | 1 | 1.5 | 2 | 5 |  | PHILIPS | SIEMENS | GE | Force |
| Yunnan Cancer Hospital | 9 | 17 | Auto 0 | | 19 0 | | 7 0 | | 0.6 | 1 | 20 | 2 | 3 |
| Sichuan Cancer Hospital | 21 | 0 | Auto 11 | | 1 0 | | 0 9 | | 0.5 | 4 | 1 | 15 | 1 |
| Shanxi Cancer Hospital | 11 | 66 | Auto 56 | | 12 9 | | 0 0 | | 0.6 | 0 | 10 | 64 | 3 |
