# Supplementary material for: Pretreatment Contrast-Enhanced Computed Tomography Radiomics for Prediction of Pathological Regression Following Neoadjuvant Chemotherapy in Locally Advanced Gastric Cancer: A Preliminary Multicenter Study
Source: Front Oncol. 2022 Jan 7;11:770758. doi: 10.3389/fonc.2021.770758 (PMC8777131; doi:10.3389/fonc.2021.770758)
Supplement: Supplementary file 3 [file Table_2.docx]

**Supplementary Table 2. Subgroup analysis of the radiomics predictive model for by sex in the training and independent external testing sets**

|  | **Male** | |  | | **Female** | |
| --- | --- | --- | --- | --- | --- | --- |
|  | Training set | Testing set |  | | Training set | Testing set |
| AUC (95% CI) | 0.962 (0.900–  1.000) | 0.815 (0.630–  0.967) | | | 0.864 (0.637-  1.000) | 0.882 (0.706-  0.941) |
| accuracy | 0.953 | 0.778 | | | 0.846 | 0.800 |
| sensitivity | 0.875 | 1.000 | | 0.605 | | 1.000 |
| specificity | 0.964 | 0.739 | | 1.000 | | 0.765 |
| PPV | 0.778 | 0.400 | | | 0.818 | 0.429 |
| NPV | 0.982 | 1.000 | | | 0.500 | 1.000 |
