# Supplementary material for: Pretreatment Contrast-Enhanced Computed Tomography Radiomics for Prediction of Pathological Regression Following Neoadjuvant Chemotherapy in Locally Advanced Gastric Cancer: A Preliminary Multicenter Study
Source: Front Oncol. 2022 Jan 7;11:770758. doi: 10.3389/fonc.2021.770758 (PMC8777131; doi:10.3389/fonc.2021.770758)
Supplement: Supplementary file 4 [file Table_3.docx]

**Supplement Table 3. Subgroup analysis of the radiomics predictive model for primary lesion site in the training and independent external testing sets**

|  | **Upper** | |  | | **Middle and lower** | | |
| --- | --- | --- | --- | --- | --- | --- | --- |
|  | Training set | Testing set |  | | Training set | | Testing set |
| AUC (95% CI) | 0.859 (0.438–1.000) | 0.857 (0.500–1.000) | | 0.892 (0.663–1.000) | | 0.812(0.639 –0.973) | |
| accuracy | 0.950 | 0.889 | | | 0.860 | | 0.658 |
| sensitivity | 0.750 | 1.000 | | | 0.833 | | 1.000 |
| specificity | 1.000 | 0.857 | | | 0.863 | | 0.606 |
| PPV | 1.000 | 0.667 | | | 0.417 | | 0.278 |
| NPV | 0.941 | 1.000 | | | 0.978 | | 1.000 |
